# The heterogeneity and associated factors of self-management behaviors in patients with type 2 diabetes: a cross-sectional latent profile analysis

**DOI:** 10.3389/fpubh.2026.1930710

**Published:** 2026-10-26

**Authors:** Chanfang Chen, Jing Su, Wenzhuan Chen, Miaolan Guo, Lichan Chen, Qingxian Liu

**Affiliations:** 1 Diabetes Education Clinic, The Second Affiliated Hospital of Shantou University Medical College, Shantou, Guangdong, China; 2 Department of Nursing, Shantou University Medical College, Shantou, Guangdong, China

**Keywords:** analysis of associated factors, diabetes self-management behaviors, latent profile analysis, illness perception, type 2 diabetes

## Abstract

**Background:**

Type 2 diabetes mellitus (T2DM) poses a growing global health burden, yet patient adherence to self-management behaviors remains suboptimal and highly variable. Traditional analyses assume homogeneity, overlooking distinct behavioral subgroups that may require different interventions. This study aimed to characterize heterogeneous subtypes of diabetes self-management behaviors among adults with T2DM using latent profile analysis (LPA), and explore clinical and psychosocial factors associated with distinct behavioral subgroups to support personalized diabetes care.

**Methods:**

In this study, 171 adults with type 2 diabetes mellitus (T2DM) were consecutively recruited from a tertiary-care hospital in Guangdong, China, between September 2024 and March 2025. Data collection included validated assessments of general demographics, diabetes self-management behaviors (Summary of Diabetes Self-Care Activities, SDSCA), illness perception (Brief Illness Perception Questionnaire, B-IPQ), diabetes health literacy (Diabetes Health Literacy Scale, DHLS), and medical coping styles (Medical Coping Modes Questionnaire, MCMQ). Latent profile analysis was conducted with Mplus 8.0 to identify distinct self-management behavioral subgroups. Multinomial logistic regression (SPSS 26.0) was used to examine correlates differentiating subgroup membership.

**Results:**

The average total SDSCA score of participants was 45.43 ± 15.79. LPA identified three interpretable latent profiles of self-management behaviors: Multidimensional Low Self-Management (31.0%, *n* = 53), Unbalanced Self-Management (13.5%, *n* = 23), and Comprehensive Optimal Self-Management (55.5%, *n* = 95). Univariate analyses detected inter-group differences in age, educational attainment, diabetic complications, diabetes duration, HbA1c, illness perception and diabetes health literacy (all *p* < 0.05). Taking the Comprehensive Optimal Self-Management group as the reference, multinomial logistic regression revealed shared correlates across two suboptimal subgroups: absence of diabetic complications and higher negative illness perception scores. Low health literacy was uniquely correlated with membership in the Multidimensional Low Self-Management profile; older age and low educational attainment (junior high school or below) were uniquely correlated with membership in the Unbalanced Self-Management profile (all *p* < 0.05).

**Conclusion:**

Adults with T2DM present prominent heterogeneity in diabetes self-management behaviors and can be divided into three distinct latent subgroups with disparate self-care patterns. Illness perception, complication status, health literacy, age and education level are variables correlated with subgroup allocation (these associations do not indicate causal effects). Targeted, individualized nursing and clinical interventions matching the characteristics of each latent profile are recommended to optimize diabetes self-management and long-term glycemic outcomes.

**Impact:**

The findings have direct implications for clinical practice by underscoring the necessity of moving beyond a ‘one-size-fits-all’ approach in diabetes care. This study highlights the need to explore the underlying features correlated with heterogeneity in self-management, which could inform the development of more effective, personalized intervention models.

**Patient or public contribution:**

The patient participants contributed by providing the survey data.

## Introduction

### Background

Diabetes mellitus constitutes a pervasive chronic metabolic disease that creates substantial clinical and public health burdens worldwide. Its pathogenesis is closely linked to various factors, including genetic susceptibility and unhealthy lifestyle exposures (1). According to epidemiological data from 2021, the global prevalence of diabetes has reached 537 million, with approximately 140 million cases in China alone (2). Type 2 diabetes mellitus (T2DM) accounts for the vast majority of these diagnoses, and national population-based surveys report a T2DM prevalence of 12.8% among Chinese adults (3). Beyond impairing patients’ physical and psychological quality of life, diabetes imposes heavy informal care burdens on family caregivers and generates enormous pressure on national healthcare systems. Projections suggest that global healthcare expenditures related to diabetes are expected to exceed 1.054 trillion USD by 2045 (4). Against this backdrop, optimizing patient’ diabetes self-management has become an indispensable core strategy to mitigate both the clinical and economic burdens of T2DM.

A growing body of evidence underscores the importance of effective self-management in enhancing the standardization of diabetes care, preventing and controlling the progression of complications, and ultimately improving patient outcomes (5). However, multiple domestic studies have demonstrated that the self-management levels of diabetes patients in China are generally at an intermediate level, with considerable variability in performance (6–8). The factors influencing self-management behaviors are diverse, and are significantly associated with variables such as age, disease duration, complications, psychological distress, social support, and family support (9, 10). Furthermore, these behaviors are also correlated with factors including disease perception, health literacy, and coping strategies (11, 12). Collectively, these observations highlight marked heterogeneity in how patients engage in diabetes self-care.

Conventional analytical approaches in prior T2DM self-management research predominantly operate under the assumption of population homogeneity. This aggregate analytic framework fails to capture inter-individual behavioral heterogeneity and conceals clinically meaningful subgroups of patients with distinct self-management patterns. By masking discrete behavioral phenotypes, such homogeneous models hinder the design of targeted, personalized intervention programs stratified by patient self-care profiles.

To fill in the research gaps, this study applied latent profile analysis (LPA) to identify heterogeneous self-management behavioral subgroups among Chinese adults living with T2DM. LPA is a statistical method that classifies individuals into latent subtypes based on their response patterns to overt variables, thereby revealing the heterogeneity of a population (13). Grouping patients with congruent self-management profiles lays an empirical foundation for tailored nursing and clinical interventions. This study further aimed to analyze the differences in characteristics and associated factors across various self-management behavior categories, with the goal of providing scientific evidence for developing personalized intervention strategies tailored to distinct sub-types.

## Materials and methods

### Aim

This study employed LPA to identify distinct self-management behavioral phenotypes among adults with T2DM, describe the unique characteristics of each latent profile, and explore correlates that differentiate these patient subgroups.

### Design

This cross-sectional observational study recruited participants from a single tertiary hospital in Guangdong Province, China, with data collection conducted between September 2024 and March 2025. Each participant completed all study assessments at one single time point. The manuscript was prepared in accordance with the Strengthening the Reporting of Observational Studies in Epidemiology (STROBE) reporting guidelines.

### Ethical consideration

Ethical approval for this study was granted by the Ethics Committee of Shantou University Medical College (approval reference number: SUMC-2022-081). All participants provided written informed consent prior to enrollment, and all study procedures complied with the principles outlined in the Declaration of Helsinki.

### Participants

Participants were recruited via convenience sampling, chosen on account of practical accessibility and logistical constraints at the study site.

Inclusion criteria: (1) Clinically diagnosed T2DM per national Chinese diagnostic criteria, with a disease duration of at least 6 months; (2) Aged 18 years or older; (3) Possessed adequate reading or verbal communication ability to finish the self-report questionnaire independently or with brief researcher guidance; (4) Voluntarily enrolled and provided written informed consent.

Exclusion criteria: (1) Severe psychiatric disorders (excluded to safeguard vulnerable groups in line with the Declaration of Helsinki); (2) Cognitive, speech, or motor impairments; (3) Critical illness, rendering participation inappropriate; (4) Inability to comply with the study procedures.

### Sample size

The study examined 25 variables, and the sample size was estimated according to the Kendall criterion, using a factor of 5 to 10 times the number of variables. Taking into account a 20% invalid response rate, the required sample size was calculated to be between 150 and 300 participants. Ultimately, a total of 178 questionnaires were collected. Of the 178 collected questionnaires, 7 were excluded: 4 due to completion time <5 min, 2 due to logical inconsistencies (contradictory answers on mutually exclusive items), and 1 due to a patterned response (identical answers for all items). After exclusion, 171 valid questionnaires were retained, with an effective response rate of 96%.

### Data collection

Five standardized measurement tools were integrated into a unified electronic questionnaire for data collection, which was hosted on the Wenjuanxing online survey platform.^1^ The opening page of the questionnaire contained clear filling guidelines and an electronic informed consent statement, and all items were set as required responses to avoid missing data.

Data collection was performed by two uniformly trained research staff via the online questionnaire. Before participants began filling out the survey, researchers elaborated on the study objectives, research significance, and standardized answering instructions. Respondents could only proceed to complete the questionnaire after confirming voluntary participation via the informed consent statement. Researchers remained on-site throughout the completion period to resolve participant inquiries and deliver real-time guidance. For participants with insufficient literacy or communication capacity to finish the questionnaire unaided, researchers read items neutrally and assisted with response recording without leading participants toward specific answers.

Rigorous data cleaning procedures were implemented to guarantee data validity and reliability. A pilot survey involving 15 adults with T2DM was conducted in advance to estimate the rational completion duration. The mean completion time was 7.2 min (SD = 1.1, median = 7.0, range: 5.5–9.5 min). Using the mean minus two standard deviations (7.2–2.2 = 5.0) as the lower bound, we set the exclusion cutoff at 5.0 min. This threshold corresponds to approximately 2 SD below the mean, ensuring that only unreasonably fast completions (likely reflecting random or inattentive responses) are excluded. Questionnaires were also discarded if they presented internal logical contradictions, obvious input errors, repetitive identical selections, or straight-lined patterned answering. All invalid records were eliminated prior to formal statistical analysis.

### Measurement

#### Sociodemographic information and medical information

Sociodemographic information of the study population included gender, age, marital status, education, and occupation status. Medical information of the study population included diabetes duration, complications, prior diabetes health education, and glycated hemoglobin (HbA1c) levels. All clinical indicators (complication status, latest HbA1c value) were extracted from the hospital electronic health record (EHR) system at the time of questionnaire administration; HbA1c data reflected laboratory results tested within 3 months before survey enrollment. Diabetes duration and history of diabetes education were self-reported by participants and cross-verified with medical records.

#### Diabetes self-management behavior

The Summary of Diabetes Self-care Activities (SDSCA) scale, developed by Toobert et al. (14) and translated and revised by Wan Qiaoqin et al. (15), is a widely used instrument to assess the self-management behaviors of diabetic patients. The scale evaluates self-care across five domains: diet, exercise, blood glucose monitoring, foot care, and medication adherence. The SDSCA consists of 11 items, with 1 item reverse-scored and the remaining 10 items scored positively. Each item is rated on an 8-point scale, ranging from 0 to 7 points. The total score ranges from 0 to 77, with higher scores indicating better self-management practices. The Cronbach’s alpha coefficient for this scale is 0.918, demonstrating high reliability and validity. It is widely applied in both clinical settings and research studies.

#### Illness perception

The Brief Illness Perception Questionnaire (B-IPQ) was developed by Broadbent et al. (16) and later translated and revised by Sun Weiming et al. (17) for use in China. This tool is designed to assess patients’ perceptions of their illness, consisting of 9 items that cover three dimensions: cognitive, emotional, and understanding. Of the 9 items, 5 are scored positively, 3 are reverse-scored, and the 9th item is an open-ended question. The remaining items are rated on an 11-point Likert scale ranging from 0 to 10, with a total score range from 0 to 80. Higher scores reflect a more negative perception of the illness. The Cronbach’s alpha coefficient for this scale is 0.831, indicating adequate reliability and validity.

#### Diabetes health literacy

The Diabetes Health Literacy Scale (DHLS) was developed by Ishikawa et al. (18) and subsequently translated by Zhao et al. (19). The scale consists of 14 items assessing three dimensions: functional health literacy, communicative health literacy, and critical health literacy. The items are rated on a 4-point Likert scale, ranging from 1 (“never”) to 4 (“most of the time”). Items within the functional health literacy dimension are reverse-scored. The total score ranges from 14 to 56, with higher scores indicating a higher level of health literacy. Cronbach’s α coefficients fall between 0.868, indicating satisfactory internal consistency.

#### Medical coping modes

The Medical Coping Modes Questionnaire (MCMQ) was developed by Feifel et al. (20) and later revised by Shen Xiaohong et al. (21). The scale consists of 20 items across three dimensions: yielding, avoidance, and confrontation. Each item is rated on a 4-point scale, resulting in a total score range from 20 to 80. The scores for each dimension reflect the respondent’s coping tendencies, with higher scores indicating a greater tendency to adopt the coping style represented by that dimension. Cronbach’s α coefficients fall between 0.64 and 0.85, demonstrating adequate reliability.

### Statistical analysis

LPA was performed with Mplus 8.0 to identify distinct subgroups exhibiting heterogeneous diabetes self-management behavioral patterns. We sequentially estimated models with 1 through 5 latent classes to select the optimal class solution.

The models were compared using Akaike Information Criterion (AIC), Bayesian Information Criterion (BIC), adjusted BIC (aBIC), entropy index, Lo–Mendell–Rubin adjusted likelihood ratio test (LMRT), and bootstrap likelihood ratio test (BLRT). Lower values of AIC, BIC, and aBIC indicated better model fit. The entropy index was used to assess the classification accuracy, and its value was in the range of 0–1; the closer it was to 1, the more accurate the classification. A significant *p*-value (*p* < 0.05) for BLRT and LMRT supported the superiority of the k-class model over the (k − 1)-class model. To ensure robustness, solutions with any latent class comprising<10% of the sample were excluded (22).

All supplementary statistical analyses were conducted in IBM SPSS Statistics 26.0. Continuous variables were summarized as mean ± standard deviation (*M* ± SD), while categorical variables were reported as counts and proportions (*n*, %). The chi-square test and one-way analysis of variance (ANOVA) were applied to compare sociodemographic, clinical, health literacy, illness perception, and coping style characteristics across the three latent T2DM self-management subgroups. Multinomial logistic regression was adopted to detect correlates differentiating the latent profiles.

Candidate predictors for the multivariable regression model were selected through a combined statistical and clinical screening strategy. Variables with *p* < 0.10 in univariate analyses were preliminarily retained. In addition, covariates with established clinical relevance and consistent associations documented in prior peer-reviewed literature were forced into the final regression model regardless of their univariate *p*-values. All statistical tests were two-tailed, and statistical significance was defined as *p* < 0.05.

## Results

### Sociodemographic and clinical characteristics of participants

A total of 171 adults with type 2 diabetes mellitus were enrolled in this study. The full sociodemographic, clinical and psychosocial characteristics of the overall sample are summarized in Table 1.

**Table 1:** General data and univariate analysis of latent profile of self-management (*n* = 171).

Variable	All (*n* = 171)	Class1 (*n* = 53)	Class2 (*n* = 23)	Class3 (*n* = 95)	Test statistic	p
Gender, *n* (%)					0.324^1^	0.850
Male	89 (52.0)	29 (54.7)	11 (47.8)	49 (51.6)		
Female	82 (48.0)	24 (45.3)	12 (52.2)	46 (48.4)		
Occupational status, *n* (%)					14.906^1^	0.061
Farmer	21 (12.3)	6 (11.3)	3 (13.0)	12 (12.6)		
Businessperson	32 (18.7)	9 (17.0)	2 (8.7)	21 (22.1)		
Worker	38 (22.2)	14 (26.4)	1 (4.3)	23 (24.2)		
Civil servant/institution staff	22 (12.9)	4 (7.5)	3 (13.0)	15 (15.8)		
Other	58 (33.9)	20 (37.7)	14 (60.9)	24 (25.3)		
Marital Status, *n* (%)					5.215^1^	0.074
Married	116 (67.8)	32 (60.4)	20 (87.0)	64 (67.4)		
Unmarried/divorced/widowed	55 (32.2)	21 (39.6)	3 (13.0)	31 (32.6)		
Educational level, *n* (%)					21.915^1^	<0.001
Junior high school or below	86 (50.3)	24 (45.3)	21 (91.3)	41 (43.2)		
Secondary vocational school or high school	38 (22.2)	17 (32.1)	1 (4.3)	20 (21.1)		
College or above	47 (27.5)	12 (22.6)	1 (4.3)	34 (35.8)		
Complications, *n* (%)					9.115^1^	0.010
Yes	114 (66.7)	37 (69.8)	9 (39.1)	68 (71.6)		
No	57 (33.3)	16 (30.2)	14 (60.9)	27 (28.4)		
Received health education, *n* (%)					4.763^1^	0.092
Yes	87 (50.9)	27 (50.9)	7 (30.4)	53 (55.8)		
No	84 (49.1)	26 (49.1)	16 (69.6)	42 (44.2)		
Age (years, *M* ± SD)	57.23 ± 9.53	56.38 ± 10.16	64.17 ± 8.26	56.02 ± 8.80	7.635^2^	0.001
Diabetes duration (years, *M* ± SD)	9.98 ± 5.97	9.64 ± 5.41	13.22 ± 7.83	9.63 ± 5.97	5.455^2^	0.005
HbA1c (%, *M* ± SD)	6.89 ± 0.60	7.14 ± 0.73	6.74 ± 0.77	6.79 ± 0.42	7.149^2^	0.001
B-IPQ (score, *M* ± SD)	45.38 ± 8.48	48.98 ± 7.54	49.09 ± 5.18	42.47 ± 8.55	14.562^2^	<0.001
HLS (score, *M* ± SD)	36.71 ± 7.88	34.75 ± 8.38	35.70 ± 7.09	38.04 ± 7.58	3.263^2^	0.041
MCMQ (score, *M* ± SD)	49.86 ± 5.22	50.79 ± 5.06	49.61 ± 6.72	49.40 ± 4.88	1.245^2^	0.291

Class 1, Multidimensional Low Self-Management; Class 2, Unbalanced Self-Management; Class 3, Comprehensive Optimal Self-Management, ^1^Chi-square (*χ*^2^) value; ^2^*F* value.

Briefly, participants had a mean age of 57.23 ± 9.53 years and a mean diabetes duration of 9.98 ± 5.97 years, with an average HbA1c level of 6.89 ± 0.60%. Half of the participants (52.0%) were male, and 66.7% had developed diabetic complications. Approximately half of the sample only received junior high school or lower education, while 50.9% had previously received diabetes health education. Mean total scores for the B-IPQ, HLS and MCMQ were 45.38 ± 8.48, 36.71 ± 7.88 and 49.86 ± 5.22, respectively.

### Latent profile analysis of diabetes self-management

Latent profile analysis (LPA) was performed on the self-management dimension scores of adults with T2DM mellitus, with sequential estimation of 1- to 5-class latent profile models. Model fit statistics for each solution are presented in Table 2. Across all models, AIC, BIC, and aBIC values declined continuously as the number of latent classes increased. The BLRT *p*-value remained statistically significant (*p* < 0.001) for all pairwise comparisons between *k*-class and (*k* − 1)-class models.

**Table 2:** Model fit statistics for latent profile analysis of diabetes self-management (*n* = 171).

Model	AIC	BIC	aBIC	Entropy	BLRT(P)	LMRT(P)	Class probability (%)
1-class	2,446.385	2,477.802	2,446.137	–	–	–	–
2-class	1,995.170	2,045.436	1,994.773	0.944	<0.01	<0.01	42.7/57.3
3-class	1,867.301	1,936.418	1,866.757	0.958	<0.01	0.0690	31.0/13.5/55.5
4-class	1,766.827	1,854.794	1,766.134	0.955	<0.01	0.2316	15.2/12.9/49.1/22.8
5-class	1,691.319	1,798.136	1,690.477	0.971	<0.01	0.0357	7.0/11.1/21.6/8.2/52.1

The 3-class solution was identified as the optimal latent profile model based on comprehensive evaluation of multiple fit indicators. First, the 3-class model yielded a high entropy value of 0.958, indicating clear discrimination between the three subgroups. Its VLMR-LRT *p*-value was 0.0690, which fell within the marginally significant range of 0.05–0.10; the VLMR-LRT test is inherently conservative, and marginal significance is acceptable in diabetes-related LPA research when supported by other favorable fit metrics. Second, the smallest subgroup of the 3-class model accounted for 13.5% of the total sample, exceeding the conventional 10% threshold to ensure stable clustering. In contrast, the 4-class model showed a reduced entropy value (0.955) and a non-significant VLMR-LRT *p*-value of 0.2316, demonstrating no meaningful improvement in classification. Although the 5-class model had the lowest information criteria and slightly higher entropy (0.971), its smallest subgroup only made up 7.0% of participants, which carried a high risk of unstable subgroup partitioning and limited clinical interpretability.

Standardized *Z*-scores were computed for subgroup profile characterization the average raw score of each SDSCA self-management dimension across the total analytical sample to facilitate direct cross-domain and cross-subgroup comparison of relative performance. Calculated *Z*-scores were reported in Table 3. A negative *Z*-score indicates the class scored below the overall sample average for that self-management domain. A *Z*-score of zero denotes performance identical to the total sample mean. A positive *Z*-score represents performance above the overall sample average.

**Table 3:** Standardized *Z*-scores of self-management dimension means across three latent profiles.

Self-management dimension	Class 1: Multidimensional Low Self-Management	Class 2: Unbalanced Self-Management	Class 3: Comprehensive Optimal Self-Management
Medication	−1.096	−0.233	0.657
Foot care	−0.735	−1.176	0.699
Glucose	−1.263	0.955	0.446
Exercise	−0.703	−1.387	0.735
Diet	−0.746	−1.059	0.675

Three qualitatively distinct self-management subgroups were distinguished based on their unique patterns of SDSCA domain performance, which we named according to their relative strengths and deficits across diet, exercise, blood glucose monitoring, foot care, and medication adherence domains. The three latent profiles were labeled as Multidimensional Low Self-Management, Unbalanced Self-Management, and Comprehensive Optimal Self-Management.

Multidimensional Low Self-Management (31.0% of sample) exhibited uniformly negative *Z*-scores across all five self-management dimensions: medication adherence (*Z* = −1.096), foot care (*Z* = −0.735), blood glucose monitoring (*Z* = −1.263), exercise (*Z* = −0.703), and diet (*Z* = −0.746). This subgroup demonstrated generally poor adherence to every core diabetes self-care task, with the most prominent deficiency in regular blood glucose testing.

Unbalanced Self-Management (13.5% of sample) displayed an uneven pattern of self-management performance. This group’s sole strength was blood glucose monitoring (*Z* = 0.955, above the sample average), whereas all remaining four domains showed subaverage scores: medication adherence (*Z* = −0.233), foot care (*Z* = −1.176), exercise (*Z* = −1.387), and diet (*Z* = −1.059). Severe deficits in physical activity, foot surveillance, and dietary control were the defining features of this profile.

Comprehensive Optimal Self-Management (55.5% of sample) achieved positive *Z*-scores across all five self-management domains, signifying consistent above-average self-care behavior. The strongest domains were exercise (*Z* = 0.735), foot care (*Z* = 0.699), and diet (*Z* = 0.675), followed by moderate strengths in medication adherence (*Z* = 0.657) and blood glucose monitoring (*Z* = 0.446). Participants in this subgroup maintained balanced, high-quality engagement in all diabetes self-management activities.

Given the relatively modest single-center sample size, all latent profile findings should be interpreted as exploratory; small subgroup size may introduce minor instability in class partitioning, which requires validation via larger multi-center cohorts in future research (Figure 1).

**Figure 1:**
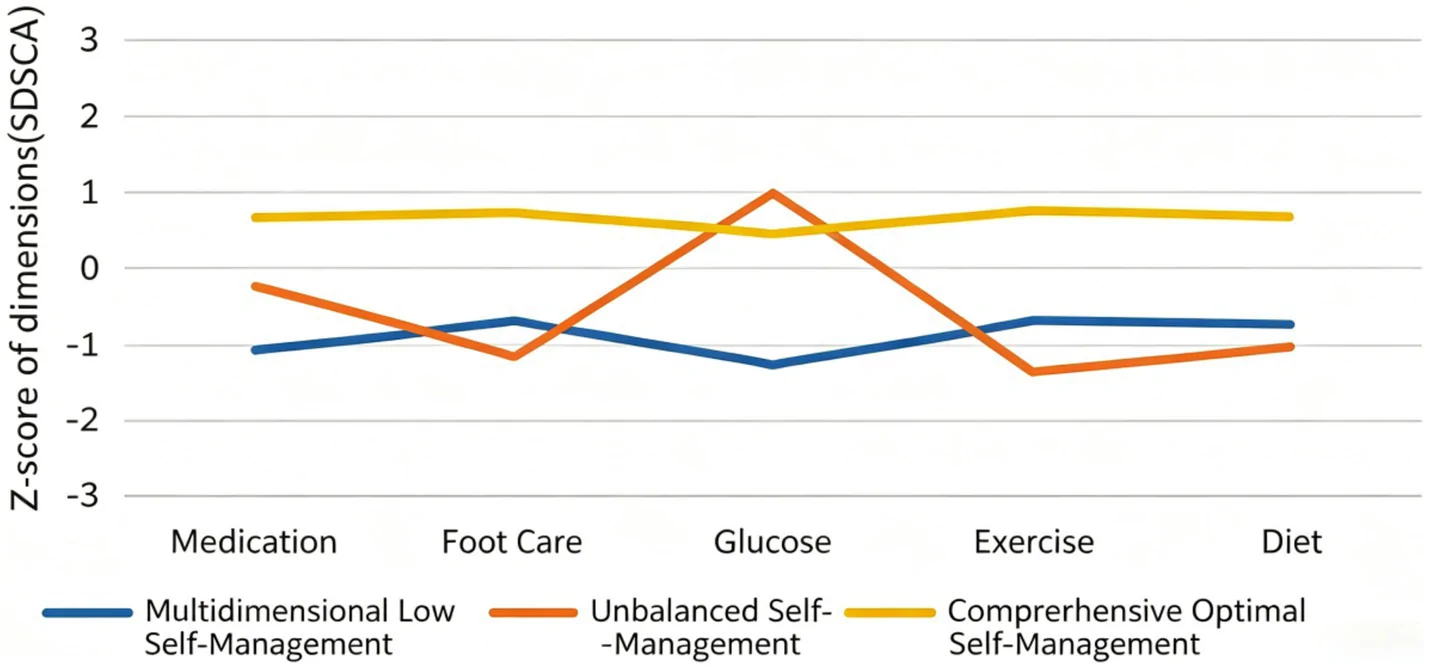
The characteristic distribution of 3 latent profiles of self-management behaviors.

### Results of univariate analysis of latent profiles of self-management in patients with T2DM

The results of univariate analysis showed that there were statistically significant differences (*p* < 0.05) among the three categories of patients in terms of education level, complications status, age, diabetes duration, HbA1c levels, and scores on the B-IPQ and HLS. Detailed results are presented in Table 1.

### Multinomial logistic regression analysis of latent profile membership

Multinomial logistic regression was performed to explore the correlates of three self-management latent profiles, with the Comprehensive Optimal Self-Management profile set as the reference group. Variables with *p* < 0.10 in univariate analyses were preliminarily retained. In addition, covariates with established clinical relevance and consistent associations documented in prior peer-reviewed literature were forced into the final regression model regardless of their univariate *p*-values. HbA1c is a downstream glycemic outcome shaped by self-management behaviors rather than a baseline covariate; thus, it was excluded from the multinomial logistic regression model to avoid circular collinearity and ambiguous interpretation of cross-sectional findings. The overall regression model was statistically significant (likelihood ratio *χ*^2^ = 106.044, df = 28, *p* < 0.001), with a Nagelkerke *R*^2^ of 0.542. The coding schemes detailed and regression outputs are presented in Tables 4, 5.

**Table 4:** Variable coding and assignment for multinomial logistic regression analysis.

Variable	Variable code	Assignment rules
Category	Y	1 = Multidimensional Low Self-Management; 2 = Unbalanced Self-Management; 3 = Comprehensive Optimal Self-Management
Occupational status	*X*1	1 = Farmer; 2 = Businessperson; 3 = Worker; 4 = Civil servant/institutional staff; 5 = Others
Marriage status	*X*2	1 = Unmarried/Divorced/Widowed; 2 = Married
Educational level	*X*3	1 = Junior high school or below; 2 = Technical secondary school or high school; 3 = College degree or above
Complications	*X*4	1 = No; 2 = Yes
Receipt of diabetes health education	X5	1 = No; 2 = Yes
Age	*X*6	Measured value
Diabetes duration	*X*7	Measured value
B-IPQ	*X*8	Measured value
HLS	X9	Measured value
MCMQ	X10	Measured value

**Table 5:** Multinomial logistic regression analysis of latent profiles of self-management in patients with T2DM (*n* = 171).

Variable	C 1 vs. C 3		C 2 vs. C 3	
	OR(95%CI)	*p*	OR(95%CI)	*p*
Educational attainment (reference: college or above)				
Junior high school or below	1.106 (0.334–3.664)	0.868	26.994 (1.565–465.637)	0.023
Secondary vocational school/high school	2.437 (0.751–7.911)	0.138	0.598 (0.073–4.889)	0.748
Marital status (reference: married)				
Unmarried/divorced/widowed	1.302 (0.563–3.012)	0.537	0.311 (0.062–1.565)	0.157
Occupational status (reference: businessperson)				
Worker	1.206 (0.349–4.168)	0.767	0.055 (0.002–1.236)	0.068
Civil servant/institutional staff	1.034 (0.199–5.380)	0.968	7.508 (0.374–150.549)	0.188
Farmer	0.877 (0.171–4.488)	0.874	0.209 (0.016–2.699)	0.230
Other occupations	2.603 (0.819–8.278)	0.105	2.672 (0.325–21.945)	0.360
Diabetic complications (reference: with complications)				
No complications	2.634 (1.002–6.925)	0.050	23.794 (4.283–132.175)	<0.001
Receipt of diabetes health education (reference: received education)				
No health education received	0.974 (0.429–2.212)	0.950	3.072 (0.650–14.511)	0.157
Age	0.992 (0.947–1.039)	0.735	1.138 (1.025–1.264)	0.016
Diabetes duration	1.018 (0.946–1.095)	0.635	1.012 (0.887–1.154)	0.864
MCMQ	1.066 (0.977–1.162)	0.150	1.009 (0.878–1.160)	0.898
B-IPQ	1.106 (1.049–1.167)	<0.001	1.130 (1.017–1.255)	0.023
HLS	0.935 (0.876–0.998)	0.045	0.974 (0.878–1.081)	0.618

C1 = Multidimensional Low Self-Managementt, C2 = Unbalanced Self-Management, C3 = Comprehensive Optimal Self-Management; the dependent variable took the C3 as the reference group. Model fit statistics: Likelihood ratio *χ*^2^ = 106.044, df = 28, *p* < 0.001; Nagelkerke *R*^2^ = 0.542.

When comparing the Multidimensional Low Self-Management profile with the reference group: Absence of diabetic complications, higher Brief Illness Perception Questionnaire (B-IPQ) scores, and lower Health Literacy Scale (HLS) scores were significantly correlated with membership in this profile (*p* < 0.05). Sociodemographic variables (age, educational level, occupation, marital status), diabetes duration, health education experience, and Medical Coping Modes Questionnaire (MCMQ) scores showed no significant correlation with profile allocation (*p* > 0.05).

When comparing the Unbalanced Self-Management profile with the reference group: Lower educational attainment (junior high school or below), older age, absence of diabetic complications, and higher B-IPQ scores were significantly correlated with belonging to the unbalanced self-management subgroup (*p* < 0.05). Occupational status (worker vs. businessperson) showed a marginally significant correlation (*p* = 0.068). Remaining sociodemographic and psychosocial variables exhibited no significant correlations (*p* > 0.05).

## Discussion

### The need for improvement in self-management behaviors among T2DM patients

The findings of this study demonstrate a moderate overall level of self-management among patients with (T2DM), as indicated by a total SDSCA score of 45.43 ± 15.79. This observation is consistent with previously published domestic studies (6, 23). Analysis across specific dimensions revealed that diet management obtained the highest score (14.93 ± 5.45 out of 28), while exercise management received the lowest (7.42 ± 4.54 out of 14). These results suggest that although patients exhibit adequate adherence to dietary recommendations, their engagement in physical activity remains insufficient. This discrepancy may be attributable to misconceptions regarding exercise-associated risks, inadequate environmental support, or physical limitations. Furthermore, suboptimal performance was observed in both blood glucose monitoring (9.96 ± 3.72 out of 14) and foot care (8.14 ± 4.40 out of 14), indicating substantial deficiencies in daily glycemic control and complication prevention. These findings are congruent with international studies that report suboptimal adherence to self-management behaviors among T2DM patients (24), highlighting the complex and multidimensional nature of diabetes self-management. Medication adherence was notably high (4.98 ± 1.29 out of 5), likely reflecting effective standardized guidance from healthcare providers and strong patient confidence in pharmacological interventions. However, excessive reliance on medication may lead to neglect of other essential self-management practices, resulting in a “pill-centric” approach that overlooks comprehensive behavioral modulation.

### Heterogeneity in self-management behaviors among adults with T2DM

Latent profile analysis (LPA) categorized the 171 participants with T2DM into three distinct behavioral subgroups: Multidimensional Low Self-Management (31.0%), Unbalanced Self-Management (13.5%), and Comprehensive Optimal Self-Management (55.5%). This three-profile classification substantiates substantial inter-individual heterogeneity in diabetes self-care engagement among Chinese adults living with T2DM, consistent with prior latent profile research focused on diabetic populations (25). The varied distribution of subgroups observed in the present study—particularly the relatively small proportion of participants falling into the unbalanced profile (13.5%)—may be shaped by the unique clinical and sociodemographic features of our single-center sample, such as varying educational attainment, duration of diabetes, and comorbidity burden.

Participants belonging to the Unbalanced Self-Management profile displayed a highly uneven pattern of self-management performance. Their only strength was blood glucose monitoring, which yielded a positive standardized *Z*-score (*Z* = 0.955) above the overall sample average; conversely, this subgroup showed pronounced deficits across exercise (*Z* = −1.387), foot care (*Z* = −1.176), diet (*Z* = −1.059), and medication adherence (*Z* = −0.233). This asymmetric self-care pattern suggests that patients in this group selectively prioritize routine glycaemic testing while neglecting long-term preventive self-care practices including physical activity, foot surveillance, and dietary regulation. Such uneven engagement may stem from overemphasis on immediate blood glucose values in clinical consultations, with insufficient provider guidance regarding holistic diabetes management.

Individuals in the Multidimensional Low Self-Management profile exhibited uniformly negative *Z*-scores across all five self-management domains, with the most severe impairment observed for blood glucose monitoring (*Z* = −1.263). This subgroup demonstrated globally inadequate adherence to every core diabetes self-care task, a pattern that may stem from inadequate diabetes-specific health literacy, negative illness perceptions, or maladaptive coping styles that hinder consistent self-care implementation across all domains.

In contrast, participants in the Comprehensive Optimal Self-Management profile obtained positive standardized *Z*-scores for medication adherence, foot care, blood glucose monitoring, exercise, and diet alike, reflecting stable, above-average performance in all self-care dimensions. This balanced, high-level self-management phenotype is plausibly supported by robust health literacy, thorough disease-related knowledge, and adaptive psychological coping resources, which enable patients to sustain comprehensive, consistent diabetes self-care behaviors. Identifying these three discrete self-management subgroups provides an empirical foundation for developing stratified, personalized nursing interventions targeted to the distinct deficits of each patient profile.

### Heterogeneity-related correlates of self-management behavior latent profiles among patients with type 2 diabetes

This cross-sectional multinomial logistic regression analysis identified distinct correlates associated with two suboptimal self-management latent profiles (Multidimensional Low Self-Management and Unbalanced Self-Management), using the Comprehensive Optimal Self-Management profile as the reference category. Consistent with recent latent profile analysis (LPA) studies examining heterogeneity in diabetes self-care, psychological illness perceptions and clinical complication status emerged as the most robust correlates of subgroup allocation, whereas most general sociodemographic factors demonstrated limited statistical association with profile membership.

#### Shared correlates across the two suboptimal self-management profiles

Both the absence of diabetic complications and elevated negative illness perception scores were significantly associated with membership in the Multidimensional Low Self-Management and Unbalanced Self-Management subgroups. This pattern aligns with a 2019 LPA study of 523 diabetes patients, which identified four latent groups based on medication-taking, diet control, exercise, and glucose-monitoring, and demonstrated that patients in the “poor medication-taking/poor lifestyle” profile exhibited consistently suboptimal self-care across multiple domains (26). Patients endorsing negative illness perceptions often adopt passive coping strategies rather than sustained multi-domain self-care—a finding corroborated by a cross-sectional study in Ghana, which demonstrated that illness perception and diabetes knowledge significantly predicted overall diabetes self-care practices, with illness perception specifically predicting exercise and diabetes knowledge predicting blood glucose testing and foot care (27). Furthermore, a 2025 LPA study among individuals with comorbid type 2 diabetes and hypertension revealed three latent profiles—"high distress,” “high health knowledge,” and “high self-efficacy”—and demonstrated that the high distress profile was characterized by greater inconsistency in medication-taking behaviors and the highest A1C levels, with racial identity and socioeconomic factors significantly associated with profile membership (28). Notably, receipt of routine universal diabetes health education showed no statistically measurable correlation with latent profile membership in the regression model. This null finding is consistent with the American Diabetes Association’s 2020 Standards of Medical Care, which recommended that diabetes self-management education and support (DSMES) be patient-centered, delivered at four critical time points (diagnosis, annually, when complicating factors arise, and when transitions in care occur), rather than as a one-time universal intervention (29).

Overall, the shared correlates identified in this study—illness perception and complication status—are broadly consistent with LPA studies on T2DM self-management heterogeneity (26, 28), as well as with the broader literature recognizing psychological representations and health-related cognitive schemas as core determinants of self-care behaviors (27, 30).

#### Unique correlates of the multidimensional low self-management profile

Lower health literacy was specifically associated with the globally poor self-management subgroup, independent of illness perception and complication status. This observation is strongly supported by a 2021 study of 524 T2DM patients, which established that health literacy positively affects self-management directly and indirectly through self-efficacy (indirect effect = 0.214, 95% CI = 0.165–0.266) and social isolation (31). A 2019 study in Iran further confirmed that functional, communicative, and critical health literacy were indirectly associated with improved glycemic control through the mediation of self-care behaviors, with critical health literacy showing the strongest effect (*β* = −0.22, *p* = 0.03) (32). A 2023 systematic review of 15 randomized controlled trials (1997–2021) concluded that health literacy–driven interventions yield positive impacts on glycemic control and self-management behaviors, with nurse-led interventions particularly effective for glycemic control and community worker-led interventions effective for diabetes knowledge and self-care (33). Individuals with insufficient health literacy struggle to interpret complex self-care requirements, leading to uniformly low adherence across all behavioral domains. In contrast to the Unbalanced Self-Management profile, neither age nor educational attainment demonstrated a significant association with this subgroup, suggesting that globally poor self-management can occur across diverse demographic strata and is primarily driven by health literacy deficits and illness cognition rather than age or educational background.

This distinctive role of health literacy, independent of demographic factors, is consistent with prior LPA research, although it diverges from studies reporting age and education as global correlates across all suboptimal profiles. This discrepancy may stem from differences in sample composition and measurement instruments, warranting further multi-center validation.

#### Unique correlates of the unbalanced self-management profile

Older age and low educational attainment (junior high school or below) were uniquely associated with the uneven self-management pattern. These demographic correlates align with a 2022 LPA study of T2DM patients in Taiwan, which categorized patients into five health profiles (C1–C5) and demonstrated that longer diabetes duration and older age were correlated with increased risk of retinopathy events, reflecting the long-term behavioral consequences of imbalanced self-management (34). A 2025 LPA study among young and middle-aged T2DM patients in China further revealed that education level, employment status, and disease duration were significantly associated with latent categories of complication risk perception, which in turn were significantly associated with self-management behavior scores (35). The marginally significant trend for occupational status (manual workers versus business professionals) must be interpreted with caution, given the small sample size of this unbalanced subgroup. Similar borderline occupational trends have been reported in small-sample single-center LPA studies and require external replication in larger, multi-center cohorts before stable conclusions can be drawn.

Notably, the finding that age and low education are uniquely associated with the Unbalanced Self-Management profile—rather than with both suboptimal profiles—represents a minor divergence from some earlier LPA studies. The 2019 Beijing LPA study found that patients in the “poor medication-taking/poor lifestyle” group were generally younger and better educated, while those in the “good medication-taking/good lifestyle” group exhibited the opposite pattern (26). This discrepancy is most likely attributable to the single-center convenience sampling design of the present study, which limits population representativeness. Future multi-center replicative LPA studies are needed to verify whether these profile-specific correlational patterns can be generalized to broader community- and hospital-based T2DM populations.

#### Clinical implications derived from the correlational findings

Based on these correlational patterns (which do not imply causality), stratified, targeted clinical strategies can be proposed for distinct self-management subgroups.

For patients matching the Multidimensional Low Self-Management profile, clinical interventions should prioritize health literacy enhancement alongside psychological counseling to alleviate negative illness perceptions. A 2020 systematic review of illness perception interventions confirmed that Common-Sense Model–based interventions can modify illness perception domains—particularly coherence, personal control, treatment control, and chronic timeline perceptions—though the evidence for glycemic control improvement remains limited (30). A 2021 randomized controlled trial further demonstrated that a Common-Sense Model–based intervention significantly improved self-care and adaptive coping among 455 T2DM patients (36). Simplified, visualized self-care guidelines and repeated skill training are recommended, as a 2023 systematic review verified that health literacy–driven interventions yield positive impacts on self-management behaviors (33).

For patients exhibiting an Unbalanced Self-Management pattern, intervention materials should be adapted for older, less educated individuals. The 2020 ADA DSMES consensus report explicitly recommends that education programs be tailored to meet the cultural, linguistic, cognitive, and literacy needs of patients, delivered through diverse settings and modalities including technology-based approaches (37). Clinicians need to emphasize the long-term risks of neglecting foot care and physical activity, rather than focusing solely on glycemic monitoring outcomes, in order to correct the selective self-care bias commonly observed in this demographic subgroup.

For all complication-free T2DM patients, routine outpatient assessments should incorporate brief illness perception screening to facilitate early risk communication, rather than waiting for complication onset to trigger self-care engagement. This screening strategy is supported by the 2025 LPA study, which demonstrated that tailored, multifaceted interventions addressing the unique challenges individuals face in managing their illnesses are crucial for improving clinical outcomes (28). The ADA’s 2020 Standards further emphasize that re-evaluation during routine care should include not only assessment of medical health but also behavioral and mental health outcomes, especially during times of deterioration in health and wellbeing (29).

Traditional universal health education programs could be restructured into profile-tailored guidance that matches the distinct behavioral barriers observed in each latent subgroup. The 2020 ADA DSMES consensus advocates for the redesign of education services to provide tailored instructions to different types of patients, based on the recognition that homogeneous interventions fail to address the heterogeneous needs of diverse patient populations (37). A 2017 LPA study in a Hong Kong primary care setting further confirmed that patients with different health-related profiles benefit differentially from empowerment-based education, with those who were older, unemployed, and weaker in self-management gaining the most from tailored sessions (38).

These clinical recommendations are grounded in the overall consistency between our correlational findings and the broader LPA literature. The minor divergence regarding the roles of age and education does not undermine the utility of profile-specific tailoring, but rather highlights the need for future multi-center studies to refine subgroup boundaries and confirm generalizability.

## Limitations

Several limitations of this study merit consideration when interpreting the results. First, data were collected from only one tertiary hospital via convenience sampling, yielding a total sample of 171 participants with the smallest latent subgroup containing just 23 patients. The modest sample size and unbalanced subgroup distribution may weaken the stability of latent profile partitioning and lower the statistical power of multinomial logistic regression. Single-center recruitment also limited demographic and socioeconomic variation within the cohort, introducing selection bias and restricting external validity. Large-scale, multi-center studies with more diverse samples are needed to replicate and verify the three self-management profiles.

Second, the cross-sectional study design cannot establish causal relationships between correlates and self-management subgroups. All detected associations, such as the correlation between glycated hemoglobin levels and profile membership, may be bidirectional. Longitudinal research is required to track temporal shifts in self-management behaviors and clarify directional effects to inform targeted interventions.

Third, all outcomes were measured through self-reported questionnaires, which fail to capture subtle contextual barriers to self-care, including time limitations, cultural beliefs about diabetes, and unrecorded life stressors. Future mixed-methods research incorporating semi-structured interviews could offer deeper insights into the root causes of divergent self-management patterns.

Finally, while this work represents one of the first domestic studies using latent profile analysis to identify T2DM self-management phenotypes, independent replications across different clinical populations are essential to confirm the generalizability of our three-subgroup classification model.

## Conclusion

Three latent self-management behavior profiles were identified among adults with type 2 diabetes mellitus, featuring distinct patterns of dietary control, exercise, blood glucose monitoring, foot care and medication adherence. Multiple demographic, clinical and psychosocial indicators correlated with patients’ subgroup membership, including illness perception, complication status, health literacy, age and education level; these correlational results do not indicate causality given the cross-sectional study design.

Noticeable heterogeneity exists in diabetes self-management among T2DM patients. Personalized, profile-specific nursing and clinical interventions rather than uniform health guidance should be implemented to improve patients’ self-management behaviors and long-term metabolic outcomes.

## Data Availability

The datasets presented in this article are not readily available because the dataset is restricted to this single study; data sharing or secondary analysis requires formal ethical approval. All participant identity information is anonymized, and any data manipulation must abide by medical privacy protection rules. Requests to access the datasets should be directed to Qingxian Liu, 15qxliu@stu.edu.cn.

## Footnotes

1
https://www.wjx.cn
